# Effect of IOP based infusion system with and without balanced phaco tip on cumulative dissipated energy and estimated fluid usage in comparison to gravity fed infusion in torsional phacoemulsification

**DOI:** 10.1186/s40662-017-0087-5

**Published:** 2017-09-17

**Authors:** Praveen K. Malik, Taru Dewan, Arun Kr Patidar, Ekta Sain

**Affiliations:** Dr Ram Manohar Lohia Hospital, New Delhi, India

**Keywords:** Active fluidics, CENTURION active, Centurion, IOP based infusion, Torsional, Phacoemulsification, Intrepid balanced tip, Kelman miniflared tip, Gravity based infusion, Cumulative dissipated energy

## Abstract

**Background:**

To evaluate the effect of three different combinations of tip designs and infusion systems in torsional phacoemulsification (INFINITI and CENTURION) in patients with cataract. According to the manufacturer, two unique improvements in the Centurion are: active fluid dynamic management system and use of an intrepid balanced tip. The study specifically aimed to evaluate the beneficial effects, if any, of change in tip design and infusion system individually and in combination on both per-operative parameters as well as endothelial health over 6 months.

**Methods:**

One hundred and twenty six consenting patients of grade 4.0–6.9 senile cataract were randomized into three groups for phacoemulsification: Group A (*n* = 42): Gravity fed infusion system and 45^0^ Kelman miniflared ABS phaco tip; Group B (*n* = 42): intraocular pressure (IOP) based infusion system and 45^0^ Kelman miniflared ABS phaco tip; Group C (*n* = 42): IOP based infusion system and 45^0^ Intrepid balanced phaco tip. The cumulative dissipated energy (CDE), estimated fluid usage (EFU) and total aspiration time (TAT) were compared peroperatively. The endothelial parameters were followed up postoperatively for six months.

**Results:**

The three arms were matched for age (*p* = 0.525), gender (*p* = 0.96) and grade of cataract (*p* = 0.177). Group C was associated with significant reductions in CDE (*p* = 0.001), EFU (*p* < 0.0005) as well as TAT (*p* = 0.001) in comparison to the other groups. All three groups had comparable baseline endothelial cell density (*p* = 0.876) and central corneal thickness (*p* = 0.561). On post-operative evaluation, although all groups were comparable till 3 months, by 6 months, the percentage losses in endothelial cell density were significantly lower in group C as compared to the other groups.

**Conclusions:**

Use of an IOP based phacoemulsification system in association with use of the Intrepid balanced tip reduces the CDE, EFU and TAT in comparison to a gravity fed system with a mini flared tip or IOP based system with a mini flared tip while also providing better endothelial preservation thus favouring the use of an IOP fed system with a balanced tip.

**Trial registration:**

Trial registration No.: CTRI/2016/06/007022.

## Background

The ultimate aim of every phacoemulsification platform is an energy efficient, time efficient yet safest possible surgical system. While an efficient phacosystem should aim at smooth, fast and well controlled phacoemulsification, minimum complication rates must accompany to complement the same. The intensity and duration of ultrasonic insult have a bearing on endothelial losses, hence the aim is to deliver the lowest energy in the eye and reduce ultrasound time simultaneously [1].

Phacoemulsification platforms have evolved to reduce intracameral ultrasound energy by introducing not only novel energy delivery techniques like micropulse, burst, torsional ultrasound, but also improvising on tip designs to have better hold, cutting efficiency and smooth aspiration [2]. However, some surgeons rank the fluidic control as having paramount importance in achieving breakthrough in phacoemulsification, attaching only secondary importance to tip designs [3].

When Centurion was launched by Alcon, the company claimed dramatic reduction of cumulative dissipated energy (CDE) in comparison to the earlier Infiniti; the claims have been verified by some independent studies [4]. The balanced tip in the Centurion has been shown to lead to lower CDE, however, a major difference also lies in the infusion system of the two platforms [5]. The superior anterior chamber stability of an intraocular pressure (IOP) fed system is hoped not only to make surgery smoother and faster, but also to protect the endothelium. Hence, to attribute all the benefit either to the balanced tip or the IOP fed infusion is not possible unless they are separately compared with identical settings for the rest of the parameters [6]. With this in mind, we designed this three arm comparative study in which first Infiniti and Centurion were compared using the same mini flared tip to find the individual effect of fluidic systems and then the miniflare tip and balanced tip were compared in the Centurion to find the specific advantage offered by this tip design.

Certain intraoperative parameters such as CDE, estimated fluid usage (EFU) and total aspiration time (TAT) can provide a comparison between any two phacoemulsification systems in terms of efficiency, but a study of the endothelium is mandatory to prove the real benefit as we treat the eye as a whole and a reduction of any factor at the cost to the endothelium is pointless. Therefore, we designed this study to compare both CDE as well as endothelial status post-operatively.

## Methods

This prospective randomized control trial was conducted in the Department of Ophthalmology, Post Graduate Institute for Medical Education and Research, Dr. Ram Manohar Lohia Hospital, New Delhi, India. The study was approved by the Institutional Ethics Committee and followed the tenets of Helsinki.

A pilot study found a mean CDE to be 30%-secs with standard deviation (SD) of ±5.5%-secs using gravity fed infusion and 45° Kelman miniflared ABS phaco tip for phacoemulsification. With a minimum number of 40 patients in each group, the study was expected to have 95% power to detect a difference of 15% in each group for phacoemulsification at 95% significance level using the student’s t-test.

Previously diagnosed patients of cataract reporting to the eye out patient department of Dr. RML hospital were approached to participate in the study. After a written informed consent, detailed history pertaining to the patient’s background, medical & ophthalmic complaints were taken. A comprehensive pre-operative eye examination was performed in each patient. Grading of cataract into grades 1.0–6.9 was done according to Lens Opacities Classification System III (LOCS III) classification. The cases with NO/NC 4.0 to 6.9 were included in the study.

Patients with pre-operative endothelial cell density (ECD) count less than 1500 cells/mm^2^, all eye pathologies that can compromise the visual recovery, eyes with any kind of corneal dystrophy or corneal scars preventing visualization of cataract for reliable grading, subluxated or dislocated lens, raised intraocular pressure (> 21 mmHg) and cases with history of previous intraocular surgery were excluded from the study. A preoperative specular microscopy using Noncontact specular microscope (EM -3000; TOMEY: VERSION 2A/OJ) was done for all cases.

One eye of each patient that fulfilled the inclusion criteria was allocated to the study. Participants were randomly distributed by an independent observer using an envelope technique into three groups: group A undergoing phacoemulsification using gravity fed infusion system and miniflared phaco tip; group B undergoing phacoemulsification using IOP based infusion system and miniflared phaco tip; and group C undergoing phacoemulsification using IOP based infusion system and balanced phaco tip.

### Surgical technique

The operations were performed by one surgeon (PKM) experienced in the technique within 1 month of recruitment. Uniform pre-regime of dilatation of the pupil was done with tropicamide (0.8%) + phenylephrine hydrochloride (5%) eye drops every 15 mins for 45 mins before surgery. Plain tropicamide was used in hypertensive patients. All surgeries were performed under topical anaesthesia or peribulbar block with 2% lidocaine + adrenaline +0.5% bupivacaine with hyaluronidase. Adrenaline was omitted in hypertensive patients. All surgeries were performed through a 2.2 mm incision. Ultrasleeves (2.2 mm) were used with Infiniti while new generation sleeves were used with the Centurion system. Following capsulorhexis, hydrodissection and rotation, the nucleus was dissembled by direct phaco chop technique. Phacoemulsification was done at 500 mmHg vacuum and aspiration flow rate of 40 cm^3^/min and 1–100% torsional phacoemulsification with IP setting at 10 ms and 1:1 ratio in all three groups. The cases were done at a bottle height of 110 cm in group A and at an IOP of 55 mmHg in groups B and C followed by irrigation and aspiration and IOL insertion.

### Per-operative outcome variables

At the time of surgery, CDE, EFU and TAT were noted in each group. Any surgical complication was also noted.

### Post-operative evaluation

Post-operative evaluation included specular microscopy at 1 week, 1, 3 and 6 months and visual acuity at 1 month after the surgery by a third observer who was neither the operating surgeon nor was aware of the surgical technique performed on the patient to avoid bias. Noncontact specular microscope (EM – 3000; TOMEY: VERSION 2A/OJ) was used to calculate ECD, coefficient of variance (CV) of cell size, percentage of hexagonal endothelial cells and corneal thickness before surgery and at each postoperative visit. Three endothelial cell photographs were taken at each visit and the mean cell count of three photographs was calculated. Endothelial cell loss was calculated as a percentage of pre-operative cell density. Visual acuity was tested using Snellen’s chart; it was converted into the logMAR scale for comparison.

### Statistical analysis

Categorical variables were presented in numbers and percentages (%) and continuous variables were presented as mean ± SD and median. Normality of data was tested using the Kolmogorov-Smirnov test. If the normality was rejected then a non-parametric test was used. Statistical tests were applied as follows:

1. Quantitative variables were compared using ANOVA/Kruskal-Wallis test (when the data sets were not normally distributed) between the three groups and ANCOVA for comparison after adjusting for confounding factors.
2. Qualitative variables were compared using Chi-Square test/Fisher’s exact test. A *p* value of <0.05 was considered statistically significant.

The data was entered in MS EXCEL spreadsheet and analysis was done using Statistical Package for Social Sciences (SPSS) version 21.0.

## Results

One hundred and twenty six eyes of 126 patients were randomized into three arms during the study.

Group A (*n* = 42): Gravity fed infusion system (INFINITY Vision System) and 45° Kelman miniflared ABS phaco tip (Alcon Laboratories, Inc., Fort Worth, TX, USA).

Group B (*n* = 42): IOP based infusion system (CENTURION Vision System) and 45° Kelman miniflared ABS phaco tip (Alcon Laboratories, Inc., Fort Worth, TX, USA).

Group C (*n* = 42): IOP based infusion system (CENTURION Vision System) and 45° Intrepid balanced phaco tip. (Alcon Laboratories, Inc., Fort Worth, TX, USA).

The demographics are demonstrated in Table 1. The three arms were matched for Age (*p* = 0.60), Gender (*p* = 0.96), Nuclear Colour (*p* = 0.17) and Nuclear Opalescence (*p* = 0.121). The nuclear grade positively correlated with age of the patient. Coaxial phacoemulsification was done in all cases. There was no incidence of posterior capsular rupture.

**Table 1:** Demographic parameters

	Group A	Group B	Group C	*p* value
Age (years)				0.525
Sample size	42	42	42	
Mean ± Stdev	62.38 ± 11.35	64.10 ± 9.57	64.76 ± 8.56	
Median	60.5	62.5	64.5	
Min-Max	42–92	42–87	50–86	
Inter quartile range	56–68	59–71	59–70	
NC (LOCSш)				0.177
Sample size	42	42	42	
Mean ± Stdev	5.34 ± 0.89	5.52 ± 0.78	5.2 ± 0.9	
Median	5.2	5.5	5.2	
Min-Max	4.0 –6.9	4.0 –6.9	4.0 –6.9	
Inter quartile range	5.0 –6.0	5.0 –6.0	4.5–5.5	
NO (LOCSш)				0.121
Sample size	42	42	42	
Mean ± Stdev	4.91 ± 0.84	5.05 ± 0.63	4.78 ± 0.87	
Median	4.7	5.0	4.7	
Min-Max	3.5–6.5	3.5–6.0	3.5–6.9	
Inter quartile range	4.5–5.5	4.5–5.5	4.0 –5.0	

*NC=* nuclear colour, *NO=* nuclear opalescence

Peroperative records of CDE, EFU and TAT were compared. The analysis revealed that a higher grade of nuclear cataract significantly correlated with higher CDE, EFU and TAT (Table 2).

**Table 2:** Correlation between nuclear grade and per-operative parameters

			NC	NO
Spearman’s rho	Age (years)	Correlation Coefficient	.379^a^	.366^a^
		Sig. (2-tailed)	.00001	.00003
		N	126	126
	CDE (%-seconds)	Correlation Coefficient	.660^a^	.643^a^
		Sig. (2-tailed)	<.0001	<.0001
		N	125	125
	TAT (seconds)	Correlation Coefficient	.619^a^	.610^a^
		Sig. (2-tailed)	<.0001	<.0001
		N	126	126
	EFU (ml)	Correlation Coefficient	.613^a^	.596^a^
		Sig. (2-tailed)	<.0001	<.0001
		N	126	126

*CDE=* cumulative dissipated energy, *TAT=* total aspiration time, *EFU=* estimated fluid usage

^a^Correlation is significant at the 0.01 level (2-tailed)

Kruskal Wallis test was applied to compare the peroperative parameters between the three groups and it was found that difference in CDE in group B when compared to group A was not statistically significant but a significantly low CDE was seen in group C in comparison to groups A and B (*p* = 0.001).

EFU was not significantly lower in group B when compared to group A, however, it was significantly lower in group C in comparison to group A as well as group B (*p* < 0.0005).

TAT was not significantly lower in group B when compared to group A, but was significantly lower in group C when compared to groups A and B (*p* = 0.001) (Table 3).

**Table 3:** Comparison of per-operative parameters between three groups

	Group A	Group B	Group C	*p* value	A vs B	A vs C	B vs C	
CDE (%-seconds)								
Sample size	42	42	42					
Mean ± SD	13.14 ± 7.65	11.33 ± 5.14	7.70 ± 4.59	0.001	0.35	0.001	0.001	Kruskal Wallis
Median	12.38	11.04	6.84					
Min-Max	1.48–28.20	2.13–20.86	1.15–16.79					
Inter quartile Range	6.40–20.80	7.02–16.21	3.69–11.94					
EFU (ml)								
Sample size	42	42	42					
Mean ± Stdev	58.19 ± 19.10	46.86 ± 12.38	34.81 ± 10.47	<.0001	0.015	<.0001	<.0001	Kruskal Wallis
Median	55.0	48.5	36.5					
Min-Max	30–92	19–74	16–56					
Inter quartile Range	40–78	39–56	26–41					
TAT (seconds)								
Sample size	42	42	42					
Mean ± Stdev	172.71 ± 94.92	131.00 ± 45.20	113.36 ± 56.50	0.001	0.299	0.0004	0.002	Kruskal Wallis
Median	132.5	125.0	95.0					
Min-Max	61–437	61–251	63–364					
Inter quartile Range	95–245	96–168	79–122					

*CDE=* cumulative dissipated energy, *EFU=* estimated fluid usage, *TAT=* total aspiration time

Thus, our results demonstrated that it is not just a change in infusion platform but a change in tip design that is associated with significant reductions in CDE, EFU as well as TAT. However, one of the main purposes of achieving such parameters is the intended safety for endothelium. Thus, we analysed the postoperative endothelial parameters as shown in (Table 4).

**Table 4:** Comparison of postoperative endothelial parameters between three groups

	Group A (n = 42)	Group B (n = 42)	Group C (n = 42)	*p* value	A vs. B	A vs. C	B vs. C	
ECD Pre-op (cell/mm^2^)				0.876	0.804	0.793	0.605	Anova
Mean ± SD	2516.00 ± 211.54	2527.17 ± 199.39	2503.86 ± 212.23					
ECD 1 week				0.712	0.959	0.499	0.463	Anova
Mean ± SD	2321.52 ± 201.18	2323.74 ± 196.11	2291.21 ± 207.79					
ECD 1 month				0.750	0.958	0.538	0.504	Anova
Mean ± SD	2227.45 ± 192.59	2229.69 ± 191.33	2200.00 ± 213.59					
ECD 3 months				0.802	0.909	0.618	0.546	Anova
Mean ± SD	2122.69 ± 192.24	2127.48 ± 191.61	2099.95 ± 223.45					
ECD 6 months				0.515	0.558	0.378	0.308	Kruskal Wallis
Mean ± SD	2007.24 ± 196.61	2012.07 ± 190.15	2005.81 ± 216.72					
CCT Pre-op (μm)				0.777	0.514	0.951	0.561	Anova
Mean ± SD	531.21 ± 25.45	526.88 ± 34.47	531.67 ± 40.52					
CCT 1 week				0.996	0.988	0.946	0.943	Anova
Mean ± SD	549.81 ± 23.32	549.71 ± 33.81	550.29 ± 39.25					
CCT 1 month				0.949	0.794	0.943	0.781	Anova
Mean ± SD	538.79 ± 24.52	537.10 ± 33.77	539.29 ± 38.11					
CCT 3 months				0.578	0.792	0.479	0.301	Kruskal Wallis
Mean ± SD	528.10 ± 24.79	523.83 ± 31.7	528.33 ± 39.39					
CCT 6 month				0.632	0.847	0.39	0.439	Kruskal Wallis
Mean ± SD	525.57 ± 24.39	524.43 ± 33.84	526.81 ± 39.55					
CV Pre-op (%)				0.022	0.017	0.017	0.832	Kruskal Wallis
Mean ± SD	39.86 ± 3.85	38.00 ± 3.78	38.07 ± 3.80					
CV 1 week				0.056	0.024	0.064	0.736	Kruskal Wallis
Mean ± SD	44.14 ± 4.22	42.14 ± 3.82	42.60 ± 4.16					
CV 1 month				0.082	0.038	0.55	0.105	Anova
Mean ± SD	39.98 ± 3.87	38.24 ± 3.67	39.50 ± 3.38					
CV 3 months				0.043	0.02	0.55	0.056	Kruskal Wallis
Mean ± SD	39.21 ± 3.58	37.43 ± 3.61	38.90 ± 3.59					
CV 6 months								Kruskal Wallis
Mean ± SD	43.17 ± 3.30	40.64 ± 3.57	42.90 ± 3.47	0.001	0.001	0.52	0.004	
6A Pre-op (%)				0.004	0.256	0.001	0.028	Kruskal Wallis
Mean ± SD	49.88 ± 6.14	48.67 ± 5.80	45.45 ± 5.60					
6A 1 week				0.051	0.569	0.02	0.073	Kruskal Wallis
Mean ± SD	32.90 ± 5.56	31.90 ± 3.76	30.17 ± 4.50					
6A 1 month				0.642	0.943	0.388	0.448	Kruskal Wallis
Mean ± SD	38.36 ± 5.64	38.14 ± 4.18	37.36 ± 4.29					
6A 3 months				0.997	0.964	0.928	0.986	Kruskal Wallis
Mean ± SD	43.93 ± 4.97	43.69 ± 3.82	43.76 ± 3.97					
6A 6 months				0.995	0.964	0.91	0.975	Kruskal Wallis
Mean ± SD	45.93 ± 4.97	45.69 ± 3.82	45.74 ± 3.96					

*ECD=* endothelial cell density, *CCT=* central corneal thickness, *CV=* coefficient of variance, *6A*= percentage of hexagonal endothelial cells

All three groups had comparable baseline ECD (*p* = 0.876) and central corneal thickness (CCT) (*p* = 0.561) but the CV was significantly lower in group B in comparison to groups A and C (*p* = 0.022) and hexagonal endothelial cell count was comparable between groups A and B but significantly lower in group C (*p* = 0.004).

Post-operative evaluation at 1 week, 1 month, 3 months and 6 months found that at all visits, ECD was in the order B > A > C and Corneal Thickness was in the order B < A < C, but none of these changes were statistically significant. The CV was in the order B < C < A at all visits with significantly lower CV in B versus A at 3 months and 6 months postop, while the hexagonal endothelial cell count was in the order A > B > C at 1 week and 1 month and A > C > B at 3 and 6 months with significantly higher counts in A vs. B only at the one week postop visit (Table 4).

As absolute values may not be representative especially when baseline values of each cornea change its susceptibility to endothelial damage, it is more relevant to study the change in parameters over time and compare between the three intervention groups. An analysis was done to compare percentage changes from baseline in endothelial parameters between the three groups at each follow up visit. Percentage changes were calculated as difference between final & baseline values divided by baseline values and multiplied by 100.

Initially, significantly lower percentage losses in ECD were seen in group A in comparison to group B as well as group C at 1 week, and in group A versus group C at 1 month. However, there was no significant difference in percentage losses in cell density among the three groups at the 3 months follow up. But by 6 months, losses were significantly lower only in group C as compared to group B. Significantly higher increase in CCT was seen in group B in comparison to both the other groups at 1 week and in comparison to group A at 1 month. However, there was no significant difference in percentage change in corneal thickness among the three groups at 3 months follow up. In all groups, CCT decreased over time but the decrease was significantly lower in group B as compared to group A at 6 months.

Significant increase in CV was seen in group C in comparison to groups A and B and in group B in comparison to group A at 1 week. The increase in CV remained significant higher in group C versus group A and B at all postoperative visits.

There was no significant difference in change in hexagonal endothelial cell count between three groups at 1 week follow up; however, the change in hexagonal endothelial cell count was significantly lower in group C in comparison to groups A and B at 1, 3 and 6 months follow up (Table 5).

**Table 5:** Percentage changes of endothelial cell density, central corneal thickness, coefficient of variance and hexagonal endothelial cells between three groups

Percentage changes	A (n = 42)	B (n = 42)	C (n = 42)	*p* value	A vs. B	A vs. C	B vs. C	
ECD 1 week				0.016	0.037	0.007	0.444	Kruskal Wallis
Mean ± SD	7.75 ± 0.84	8.08 ± 1.03	8.54 ± 1.47					
ECD 1 month				0.071	0.18	0.031	0.264	Anova
Mean ± SD	11.48 ± 0.85	11.81 ± 1.32	12.22 ± 2.00					
ECD 3 months				0.634	0.352	0.491	0.876	Kruskal Wallis
Mean ± SD	15.66 ± 1.53	15.88 ± 1.43	16.29 ± 3.24					
ECD 6 months				0.061	0.671	0.069	0.027	Kruskal Wallis
Mean ± SD	20.31 ± 1.29	20.47 ± 1.53	20.06 ± 3.06					
CCT 1 week				0.006	0.001	0.379	0.038	Kruskal Wallis
Mean ± SD	−3.62 ± 1.36	−4.36 ± 0.84	−3.57 ± 2.27					
CCT 1 month				0.089	0.016	0.737	0.096	Anova
Mean ± SD	−1.38 ± 1.23	−1.96 ± 0.88	−1.49 ± 1.58					
CCT 3 months				0.205	0.114	0.985	0.135	Kruskal Wallis
Mean ± SD	0.63 ± 1.33	0.51 ± 2.83	0.61 ± 1.41					
CCT 6 months				0.110	0.033	0.648	0.17	Kruskal Wallis
Mean ± SD	1.05 ± 1.32	0.46 ± 0.86	0.9 ± 1.42					
CV 1 week				<.0001	0.041	0.0001	0.003	Kruskal Wallis
Mean ± SD	−10.81 ± 3.11	−11.00 ± 1.45	−11.99 ± 4.46					
CV 1 month				<.0001	0.166	<.0001	<.0001	Kruskal Wallis
Mean ± SD	−0.34 ± 2.77	−0.69 ± 2.84	−3.97 ± 4.63					
CV 3 months				<.0001	0.762	<.0001	<.0001	Kruskal Wallis
Mean ± SD	1.54 ± 2.35	1.45 ± 2.45	−2.34 ± 4.54					
CV 6 months				<.0001	0.055	<.0001	<.0001	Anova
Mean ± SD	−8.55 ± 3.71	−7.11 ± 3.07	−12.98 ± 5.16					
6A 1 week				0.803	0.926	0.63	0.534	Anova
Mean ± SD	34.19 ± 5.59	34.29 ± 4.28	33.57 ± 6.12					
6A 1 month				0.002	0.308	0.001	0.005	Kruskal Wallis
Mean ± SD	23.04 ± 6.46	21.32 ± 5.67	17.32 ± 8.67					
6A 3 months				<.0001	0.534	<.0001	0.0004	Kruskal Wallis
Mean ± SD	11.43 ± 7.85	9.65 ± 7.11	2.96 ± 9.54					
6A 6 months				<.0001	0.274	<.0001	0.0004	Anova
Mean ± SD	7.36 ± 8.15	5.48 ± 7.49	−1.62 ± 9.95					

*ECD=* endothelial cell density, *CCT=* central corneal thickness, *CV=* coefficient of variance, *6A*= percentage of hexagonal endothelial cells

As age and nuclear grade can affect endothelial cell loss, we asked if there were any endothelial salvaging effects with respect to the phacoemulsification system used. An ANCOVA test was performed to compare changes in the endothelium after adjusting for these confounders. Interestingly, even after adjusting for these variables, the results remained significant (Table 6).

**Table 6:** Percentage changes of endothelial cell density, central corneal thickness, coefficient of variance and hexagonal endothelial cells between three groups after adjusting for age, NC, and NO

Percentage Changes	After adjusting for age, NC, NO		
	A vs. B	A vs. C	B vs. C
ECD 1 week	0.109	0.003	0.154
Mean ± SD			
ECD 1 month	0.155	0.017	0.453
Mean ± SD			
ECD 3 months	0.399	0.249	0.559
Mean ± SD			
ECD 6 months	0.402	0.738	0.315
Mean ± SD			
CCT 1 week	0.002	0.705	0.006
Mean ± SD			
CCT 1 month	0.011	0.859	0.015
Mean ± SD			
CCT 3 months	0.821	0.979	0.65
Mean ± SD			
CCT 6 months	0.021	0.652	0.033
Mean ± SD			
CV 1 week	0.646	0.254	0.161
Mean ± SD			
CV 1 month	0.556	0.0001	0.0003
Mean ± SD			
CV 3 months	0.874	<.0001	<.0001
Mean ± SD			
CV 6 months	0.069	<.0001	<.0001
Mean ± SD			
6A 1 week	0.998	0.478	0.577
Mean ± SD			
6A 1 month	0.214	0.001	0.031
Mean ± SD			
6A 3 months	0.291	0.0001	0.001
Mean ± SD			
6A 6 months	0.291	<.0001	0.001
Mean ± SD			

*ECD=* endothelial cell density, *CCT=* central corneal thickness, *CV*= coefficient of variance, *6A=* percentage of hexagonal endothelial cells

## Discussion

A lower CDE is one parameter that all surgeons vie to achieve due to the obvious benefits of reduced energy delivery, which correlates with less damage to the endothelium and lower complication rates [7]. Torsional phacoemulsification reduces energy delivery in the eye in comparison to longitudinal phacoemulsification [8]. In addition, better fluidic management increases the efficiency of phacoemulsification to result in lower energy delivery and at the same time, a stable anterior chamber correlates with lower endothelial cell loss [9].

While previous machines have traditionally used gravity fed infusion systems, some drawbacks were apparent [10]. An attempt was made to use more stable modalities like an air pump or pressurised irrigation bottle with gas with some benefits. However, as the gas infusion pressure does not necessarily vary in response to the changing aspiration flow rate, the effect is the same as raising the bottle height [4, 11]. More recent machines maintain IOP by using a compliant irrigation bag, which is squeezed in response to the aspiration flow rate and estimated incision leakage. Centurion uses an Active Fluidics Technology as compared to a gravity fed infusion system in the Infiniti [4].

Instead of a bag or bottle suspended above the machine to use gravity as just described, there is a compartment into which a bag of balanced salt solution (BSS) (Alcon Laboratories, Inc.) is placed between two paddles. The bag is made of a polymer which, under the pressures to which it is subjected, is almost stretch-free. This means that very small and precise movements of the electronically controlled paddles against the bag are designed to lead to exquisite control of the fluid entering the eye. This control is part of the technology called ACTIVE FLUIDICS, which allows the surgeon to choose a target IOP. To assist in the way the paddles move, the outflow and inflow from the CENTURION Vision System are monitored by pressure sensors on the Fluidics Management System (FMS). To speed the responsiveness and thus rise time, there are two rotary valves. The pump itself now has seven rollers, compared with four on the INFINITI Vision System (Alcon Laboratories, Inc.). These seven rollers act on the entire 360° of the tubing in which they are in contact, compared with only 180° on the INFINITI Vision System. This again is designed to lead to improved response times. The aspiration tubing has a reduced inner diameter of 0.048 mm in comparison to 0.057 mm of the Intrepid plus FMS being used in the INFINITI and is also very difficult to compress. This is designed to have two effects: firstly, to increase resistance to flow; and second, to prevent any tubing from collapsing, which can reduce post-occlusion surge and thus increase anterior chamber stability. This system therefore provides an active control of infusion pressure to maintain a more stable target IOP level in spite of variations in aspiration flow rates [12, 13].

An experimental study with the Infiniti versus Centurion Gravity versus Centurion Active has shown better IOP maintenance and thus surge protection with Centurion Active as compared to other gravity fed systems at increasing aspiration flow rates. However, in view of an artificial anterior chamber used in the study, it was recommended that comparative studies are performed using the tissue globe environment [4, 5, 12].

Previous studies have shown lowered CDE in the Centurion as compared to the Infiniti [4, 14–16]. According to the manufacturer, two unique improvements in the Centurion are: active fluid dynamic management system and use of an Intrepid balanced tip [17].

Apart from the surgeon’s skill and technique, use of torsional phaco instead of longitudinal can have further lowered CDE and fluid usage if the cutting efficiency of the tip is maximized. If we try to visualize the tip movement in the lens during torsional phacoemulsification, two things gain importance. First, an increasing bevel exposes a greater cross-sectional area to lens matter, increasing its cutting efficiency. Secondly, the bend in the shaft of the tip increases the area it traverses on each stroke so the 22-degree bent 30-degree Kelman mini-flared tip cuts longer than the 12-degree bent 30-degree mini-flared Kelman tip [18, 19].

Design of phacotips for torsional phacoemulsification has evolved over time specially to get the best out of torsional mode of phacoemulsification. The Kelman style angled tip was followed by mini flared ABS Kelman tip and then the 45-degree tip, which has significantly better cutting action and repositioning of the lens material than the 30-degree bevelled tip [18, 20–22]. Lower CDE and less CCT changes have been observed with the 45-degree aperture angled tip than with the 30-degree aperture angled tip [23].

However, the Kelman tips have significant motion of the shaft as well, which can lead to heat production and undesired corneal changes at the incision. The Intrepid balanced tip has enhanced sideways displacement at the tip end and greatly reduced tip action along the shaft. It has a stroke length of 192 μm at 100% OZIL as compared to 130 μm at 100% OZIL with 45° Kelman miniflare ABS. Lower CDE and no wound changes are attributed to the increased emulsification and cavitation at the sides of the INTREPID balanced tip but no significant phacotip shaft motion was seen with the balanced tip [8, 24]. An independent comparative study of the designs of the 45° Kelman and 45° balanced phacotip showed less total US time, torsional time, CDE and BSS use as well as similar changes in the ECD with a balanced tip [25].

Similar results were reported using these two tip designs in the CENTURION in patients belonging to the same ethnic group as us [26]. In our study, we too found that the use of the Intrepid balanced tip in combination with Active Fluidics was associated with significant reduction in CDE, EFU as well as TAT in comparison to the other groups using a 45° Kelman tip on either a gravity fed or IOP fed fluidic system.

The reduced energy delivery and stable IOP should also translate to healthier corneas. There exists a relation between ultrasound power and corneal endothelium with increased energy usage being associated with endothelial cell loss. CDE is only one way of expressing ultrasonic energy usage. CDE is an estimation of the energy at the incision site experienced during the removal of cataractous lens and is measured in %-secs. The incision is defined as 5.6 mm back from the cutting edge of the tip. A lower CDE indicates that less energy was present at the incision site [27]. This should ideally reflect higher incision burn with increased CDE [28].

But what we are interested in is not just the effect on the endothelium at the incision site but the overall health of the entire and more usefully central endothelium. Comparing CDE with tip designs varying in length may not exactly be representative as ideal comparisons and we might end up comparing apples to oranges instead of apples to apples [6]. Also, reduced CDE even though good at incision may not always result in reduced energy delivery in the anterior chamber if greater tip excursion and cavitation are being achieved [9].

This justifies choosing technical modifications that have equivalent or safer endothelial results if other benefits of reduced ultrasound time, EFU and in addition, CDE are present. A previous study comparing 2 tip designs had revealed that balanced tip use is associated with less total ultrasound time, torsional time, CDE and BSS use with similar changes in ECD as with the Kelman tip.

In our study, there was a direct correlation between CDE and the nuclear grade indicating that higher grades of cataract required more phaco energy. Similar results were shown in a study by Liu et al. where increase in CDE was seen with higher grades of nuclear density [29]. We also saw that higher grades of cataract correlated significantly with increases in CDE, EFU and TAT. Thus, the changes in endothelial status were compared across three groups after adjusting for any possible effect of age and nuclear grade and found that at 6 months, changes in ECD were comparable among 3 groups with group B showing greater increase in CCT. Although group C had significantly higher increase in CV than the other two groups, it had better preservation of hexagonal endothelial cell counts. This suggested comparable endothelial safety between three groups with added advantage of lowered CDE, EFU and TAT in the Centurion with an Intrepid balanced tip.

Even if we were to correlate CDE with incision temperature only, we cannot ignore the lowered EFU as well as TAT with the use of Centurion in the setting of comparably safe endothelium even up to six months follow up. Thus, we recommend using the Centurion system with the Intrepid balanced tip to obtain its maximum benefit in terms of reduction in CDE, EFU and TAT.

## Conclusions

Use of an IOP based phacoemulsification system in association with the use of an Intrepid balanced tip provides comparable endothelial results as well as reduces the CDE, EFU and TAT versus a gravity fed system with mini flare tip or IOP based system with mini flare tip.

## Abbreviations

6A: Hexagonal endothelial cells
BSS: Balanced salt solution
CCT: Central corneal thickness
CDE: Cumulative dissipated energy
CV: Coefficient of variance
EFU: Estimated fluid usage
IOP: Intraocular pressure
LOCS III: Lens opacities classification system ш
NC: Nuclear colour
NO: Nuclear opalescence ECD Endothelial cell density
OPD: Out patient department
SD: Standard deviation
TAT: Total aspiration time
